# Evidence of a Muscle–Brain Axis by Quantification of the Neurotrophic Myokine METRNL (Meteorin-Like Protein) in Human Cerebrospinal Fluid and Serum

**DOI:** 10.3390/jcm10153271

**Published:** 2021-07-24

**Authors:** Martin Berghoff, Alexandra Höpfinger, Ranjithkumar Rajendran, Thomas Karrasch, Andreas Schmid, Andreas Schäffler

**Affiliations:** 1 Department of Neurology, Giessen University Hospital, 35392 Giessen, Germany; martin.berghoff@neuro.med.uni-giessen.de (M.B.); Ranjithkumar.Rajendran@neuro.med.uni-giessen.de (R.R.); 2 Department of Internal Medicine III, Giessen University Hospital, 35392 Giessen, Germany; alexandra.hoepfinger@innere.med.uni-giessen.de (A.H.); thomas.karrasch@innere.med.uni-giessen.de (T.K.); andreas.schaeffler@innere.med.uni-giessen.de (A.S.)

**Keywords:** myokine, METRNL (Meteorin-like protein), cerebrospinal fluid, blood–brain barrier, adipokine

## Abstract

Data on the quantification of the potentially neurotrophic adipo-myokine METRNL (Meteorin-like protein) in human cerebrospinal fluid (CSF) are lacking and migration of this secreted protein across the blood–brain barrier (BBB) is uncertain. In the present pilot study, METRNL concentrations were quantified by ELISA in paired serum and CSF samples of 260 patients (107 males, 153 females) undergoing neurological evaluation. METRNL was abundant in serum (801.2 ± 378.3 pg/mL) and CSF (1007.2 ± 624.2 pg/mL) with a CSF/serum ratio of 1.4 ± 0.8. Serum METRNL levels were significantly correlated (rho = +0.521) to those in CSF. CSF METRNL concentrations were significantly correlated (rho = +0.480) with albumin CSF/serum ratios. The CSF/serum ratios of METRNL and albumin were positively correlated in Reibergram analysis (rho = 0.498), indicating that raising CSF concentrations of METRNL are mediated by increasing BBB dysfunction. The CSF concentrations of METRNL strongly increased in a stepwise manner along with increasing BBB dysfunction from grade 0 to grade 3 and with rising CSF cell count. CSF/serum ratio of METRNL also increased from grade 0 (1.2 ± 0.7) to grade 3 (3.0 ± 0.2). Furthermore, CSF levels were positively correlated with age. In conclusion, METRNL is a secreted and neurotrophic myokine that crosses over the BBB. CSF concentrations of METRNL increase with BBB dysfunction.

## 1. Introduction

The hypothesis of a “fat–brain axis” [1,2] is that adipocyte-specific secretory hormones, proteins and peptides are released by white adipocytes (“*adipokines*”) or brown adipocytes (“*batokines*”*)* into the circulation, cross over the blood–brain barrier (BBB), appear in cerebrospinal fluid (CSF) and finally regulate central nervous system functions such as appetite, satiety, sympathetic neural outflow, temperature, energy expenditure, pituitary and hypothalamic function, stress reaction, energy appeal reaction and others [3,4,5]. The very first identified and most prominent mediator of the *fat–brain axis* is represented by the satiety hormone leptin [2]. During the past decades, several other adipokines [1,6,7,8] such as adiponectin, resistin, progranulin, adipsin, CTRP-3, RBP4, clusterin, and PEDF have been detected in CSF and shown to cross over the BBB.

Similarly, the concept of a *muscle–brain axis* [9,10] has been discussed recently due to the fact that myocytes represent secretory cells releasing “*myokines*” [9,10,11] or “*adipo-myokines*” such as myonectin, myostatin, IL-6, IL-8, IL-15, FGF-21, irisin, and sclerostin [12]. The muscle secretome probably consists of several hundred secreted peptides [11,12], many of them being upregulated and released upon muscle contraction [10,13,14]. Exercise-induced release of lipolytic myokines such as irisin and IL-6 increase thermogenesis via browning of adipose tissue [10].

Meteorin-like protein(METRNL; also named subfatin or IL-41) has been discussed as a novel adipo-myokine secreted by adipocytes and muscle cells [15,16]. Exercise and cold exposure increases myocyte METRNL expression, which then upregulates energy expenditure, induces thermogenesis, improves glucose tolerance, exerts anti-inflammatory effects by alternative activation of monocytes, and directs eosinophils into adipose tissue [16,17,18,19]. Anti-inflammatory and thermogenetic actions of METRNL are mediated indirectly via eosinophil-dependent increase in IL-4 expression [16]. Treatment of mice with exogenous METRNL alleviates inflammatory processes and insulin resistance and upregulates fatty acid oxidation via AMPK (adenosine monophosphate-activated kinase) and PPARδ signalling pathways [20]. METRNL^-/-^ mice are viable, but have a dysregulated cytokine production and are highly susceptible to LPS in a sepsis model [21].

Interestingly, METRNL has been suggested to act as a neurotrophic factor with therapeutic potential in neural development [22]. However, METRNL has not yet been measured in CSF of mice/rodents or humans and it is completely unknown whether the secreted protein is able to cross over the BBB. Thus, the detection and systematic quantification of significant protein concentrations of METRNL in CSF might provide the basis for novel physiological and pathophysiological insights into a postulated *muscle–brain axis* with a translational impact (drug targets, biomarkers).

It was the aim of the present study:

- to quantify the protein concentrations of the neurotrophic adipo-myokine METRNL by ELISA in paired serum and CSF samples from a well- characterized cohort of patients (*n* = 260) with various diseases who underwent neurological evaluation, including lumbar puncture in a single and tertiary care centre;
- to correlate serum/CSF METRNL levels with anthropometric parameters, routine laboratory parameters and neurological and internal medicine diseases;
- to obtain insight into the basal and inducible regulation of BBB function with respect to the migration of METRNL by providing novel and specific CSF/serum ratios and classical *Reibergrams*;
- to provide evidence whether or not METRNL fulfils the biochemical and physiological conditions to act as a mediator of the *muscle–brain axis*.

## 2. Materials and Methods

### 2.1. Study Population

Serum and CSF samples were collected from patients undergoing neurological evaluation at the Department of Neurology, University Hospital of Giessen, Germany. General patient characteristics and routine laboratory parameters were published earlier by our group in studies on the role of several adipokines [1,6,7,8] in serum and CSF. Briefly, serum was prepared from whole blood by centrifugation (4 °C, 10 min, 4000 rpm). Serum and CSF samples were immediately stored at −20 °C. All participants were informed about the aim of the study and gave informed consent. The study was approved by the local ethical committee (registration code AZ 287/13). The exclusion criteria were pregnancy and age < 18 years. Anthropometric and patient-related data such as age, gender, body mass index (BMI), underlying diseases and medication were recorded. The occurrence of type 1 or type 2 diabetes mellitus, dyslipidaemia, coronary artery disease, and thyroid disease was documented. Smoking habits and medication, especially the use of levothyroxine, statins and hormonal contraceptives were also recorded. The diagnosis was made by a board-certified neurologist. Patients were divided into seven subgroups for evaluation: (1) infectious diseases of the central nervous system (CNS), (2) multiple sclerosis, (3) vascular diseases (brain and spinal infarction), (4) epilepsy, (5) headache/facial pain, (6) neuropathy/cranial nerve palsy, (7) others (consisting of dementia, psychiatric disorders, normal pressure hydrocephalus (NPH), rare diseases such as amyotrophic lateral sclerosis, and patients undergoing spinal puncture for exclusion of other diseases). Based on their CSF/serum albumin ratio, patients were divided into 4 subgroups: grade 0: ratio < 6.5 × 10^−3^ for age < 40 years and <8.0 × 10^−3^ for age < 60 years; grade 1: <10.0 × 10^−3^; grade 2: >10.0 × 10^−3^; grade 3: >20.0 × 10^−3^. According to their CSF cell count, patients were divided into 2 subgroups: 0–5 cells/μL and >5 cells/μL.

### 2.2. Measurement of CSF and Serum Parameters

Red and white blood cell count, serum total protein, serum albumin, serum creatinine, C-reactive protein, serum glucose, AST, ALT, uric acid, total bilirubin, cholinesterase, glycosylated haemoglobin concentration (HbA1c) and lipid parameters (triglycerides, cholesterol, HDL cholesterol, LDL cholesterol) were measured by standard methods at the Institute of Clinical Chemistry and Laboratory Medicine, University Hospital of Giessen, Germany. Total cell count, total protein, albumin, glucose, lactate, ferritin, and immunoglobulins G, A, and M were measured in serum and CSF by using standard techniques in the Neurochemical laboratory of the Department of Neurology, University Hospital of Giessen, Germany. The basal anthropometric and laboratory characteristics were published earlier by our group [1,6,7,8]. CSF/serum ratios were calculated for albumin and METRNL and were plotted as in typical *Reibergrams*.

### 2.3. High-Sensitive Quantification of METRNL in Serum and CSF

The collected paired samples of human serum and cerebrospinal fluid were stored at −20 °C in aliquots of 150 µL. Serum and CSF levels of METRNL were measured in duplicate by high-sensitive enzyme-linked immunosorbent assays (ELISA) purchased from R & D Systems, Wiesbaden, Germany (Human Meteorin-like/METRNL DuoSet ELISA). The assay range was 15.6–1000 pg/mL.

### 2.4. Statistics

For statistical analysis, data were exported into the statistical software program *SPSS 27.0*. METRNL serum concentrations did not follow a *Gaussian* distribution. Non-parametric numerical parameters were analyzed by *Mann–Whitney U*-test (for 2 unrelated samples) and *Kruskal–Wallis* test (>2 unrelated samples). Correlation analysis was performed by using the *Spearman rho* test (non-parametric parameters). Partial correlation analysis was applied to control for possible covariates. A *p*-value below 0.05 (two-tailed) was considered as statistically significant.

## 3. Results

### 3.1. Characteristics of the Study Population and Concentrations of METRNL in Serum and CSF

A total of 260 patients, 107 males (41.2%) and 153 females (58.8%), were included (mean age 50.5 ± 17.5 years) in the study (Table 1). Of the sample, 48.5% were of normal weight and 49.2% were overweight/obese (BMI ≥ 25 kg/m²) (BMI was not available in 2.3%). Of the patients, 11.5% were diagnosed with diabetes mellitus (type 1 or 2).

We were able to detect METRNL by ELISA both in the serum and CSF. The concentrations of METRNL could successfully be measured in serum and CSF of all patients in duplicate. In serum, METRNL concentrations ranged from 203.1 pg/mL to 2685.1 pg/mL. The mean value was 801.2 pg/mL with a standard deviation of 378.3 pg/mL. In CSF, METRNL ranged from 230.0 to 4275.3 pg/mL. The mean CSF concentration was similar to the respective serum levels with 1007.2 ± 624.2 pg/mL. For the first time in the literature, we are able to provide data that are suitable to calculate a specific CSF/serum ratio for METRNL. This ratio ranged from 0.2 to 6.7 (mean: 1.4 ± 0.8) (Table 1).

Male patients had significantly lower METRNL concentrations in serum (745.3 pg/mL ± 350.9 pg/mL) (*p* = 0.031), whereas there was a non-significant trend (*p* = 0.052) to higher METRNL concentrations in CSF (1071.2 pg/mL ± 603.4 pg/mL) when compared to females (serum: 840.2 pg/mL ± 392.8 pg/mL, CSF: 962.4 pg/mL ± 636.5 pg/mL). Patients with hypertension had significantly higher METRNL concentrations in CSF (1117.5 pg/mL ± 673.1 pg/mL) than normotensive subjects (929.6 pg/mL ± 575.2 pg/mL) (*p* = 0.023) (Table 1). There was no significant difference in serum or CSF METRNL concentrations concerning patients with/without diabetes mellitus, normal weight versus overweight/obese patients, and smokers versus non-smokers (Table 1).

### 3.2. Correlation of Serum and CSF METRNL Concentrations with Numerical Standard Variables

Table 2 (left column) summarizes the correlations of serum METRNL concentrations with standard serum and CSF parameters. Table 2 (right column) depicts the correlations of CSF METRNL concentrations with serum and CSF parameters. METRNL concentrations in serum showed a significantly positive correlation with the inflammatory parameter CRP (rho = +0.159, *p* = 0.011) but not with leukocyte count. METRNL concentrations in CSF were correlated significantly and positively with several markers of BBB dysfunction (other than albumin ratio) such as total protein (rho = +0.420, *p* < 0.001), albumin (rho = +0.463, *p* < 0.001), immunoglobulins (IgM (rho = +0.349, *p* = 0.003), IgG (rho = +0.357, *p* < 0.001), IgA (rho = +0.430, *p* < 0.001)), and lactate (rho = +0.210, *p* = 0.001).

### 3.3. CSF Concentrations and CSF/Serum Ratios of METRNL Significantly Increase with Biochemical Parameters Blood–Brain Barrier (BBB) Dysfunction

METRNL concentrations in CSF (*p* < 0.001) significantly increased with increasing BBB dysfunction in a stepwise manner from grade 0 to grade 3 (Table 3). Accordingly, the calculated CSF/serum METRNL ratio also rose significantly (*p* < 0.001) in a stepwise manner along with increasing blood–brain barrier (BBB) dysfunction. There was no significant difference between the subgroups of neurological disease entities. These data indicate, for the first time, that CSF METRNL concentrations are dependent on the integrity of the BBB and that leakage of the BBB increases the respective CSF concentrations. Furthermore, higher counts of CSF immune cells were associated with elevated CSF and serum METRNL concentrations (Table 3). In this context, it is worthwhile to remember that serum METRNL concentrations were shown to correlate to systemic CRP levels. The presence of oligoclonal bands (a putative marker of multiple sclerosis) was not associated with serum and CSF METRNL concentrations.

### 3.4. Detailed Evaluation of Classical Reibergrams for the Quantitative Description of BBB Dysfunction

**Figure 1:**
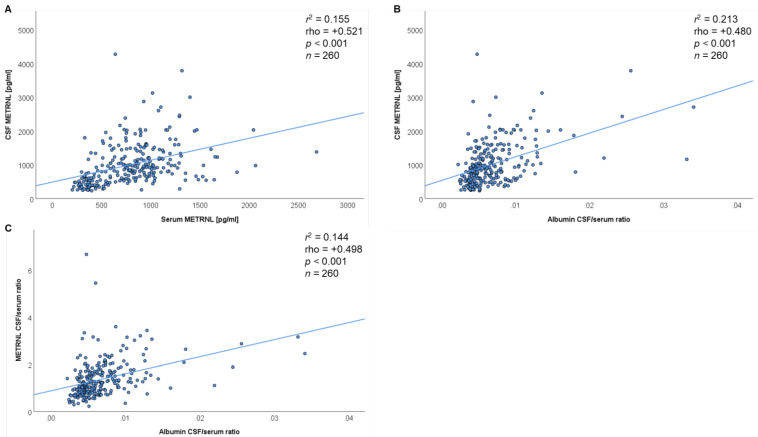
Scatterplot diagrams and Reibergrams indicating the migration of METRNL across the blood–brain barrier. (**A**) Positive correlation between METRNL serum and CSF concentrations. (**B**) Positive correlation between albumin CSF/serum ratios and CSF METRNL concentrations. (**C**) Positive correlation between albumin CSF/serum ratios and METRNL CSF/serum ratios (Reibergram). The *Spearman rho* test was used.

Figure 1A summarizes the respective serum–CSF correlations of METRNL by scatterplots and according to the *Spearman rho* test. The serum METRNL concentrations were significantly (*p* < 0.001) correlated (rho = +0.521) with the respective CSF concentrations. This indicates that individuals with higher serum METRNL levels overall have a significant trend to higher CSF concentrations. In more detail, the calculated albumin CSF/serum ratio (as a common and widely used predictor of BBB permeability) was significantly associated with CSF METRNL concentrations (rho = +0.480; *p* < 0.001) (Figure 1B). This indicates that increasing BBB dysfunction causes higher CSF METRNL levels. Similar to classical *Reibergrams*, we then plotted the CSF/serum ratios for METRNL against the CSF/serum ratios for albumin (Figure 1C). The CSF/serum albumin ratio was significantly and positively correlated with the CSF/serum ratio of METRNL (*p* < 0.001; rho = +0.498). This finally supports the role of BBB permeability for the migration of METRNL over the BBB into the central nervous system. Since BBB permeability increases with age, the observed correlation between age and CSF METRNL concentrations is reasonable and relevant from a clinical point of view.

### 3.5. Correlation of Serum and CSF METRNL Levels with Respect to the Metabolic Syndrome Complex

Adipo-myokines are of physiological relevance in the context of the metabolic syndrome (MetS). The MetS is characterized by obesity, high triglycerides and low HDL cholesterol, hypertension, and insulin resistance/type 2 diabetes mellitus. Since these clinical parameters were available in the patients, we tested whether METRNL was correlated to single entities of the MetS. Neither serum nor CSF METRNL levels were correlated to BMI, triglycerides, HDL cholesterol, LDL cholesterol, or the occurrence of type 2 diabetes mellitus. As mentioned above (Table 1), CSF METRNL levels were higher in hypertensive versus normotensive individuals; however, the meaning of this descriptive observation remains elusive.

## 4. Discussion

The present data clearly indicate for the first time that there exists a very high grade of both basal and inducible permeability of the blood–brain barrier (BBB) for METRNL. The basal permeability was proven by the highly significant correlation between serum and CSF METRNL concentrations and by specific calculation of the relatively high CSF/serum ratio of 1.4 ± 0.8. When compared to other adipokines [1,6,7,8], this ratio was extensively (~100- to 1000-fold) higher than ratios calculated for adiponectin (0.7 × 10^−3^), resistin (4.4 × 10^−3^), adipsin (27 × 10^−3^), RBP4 (8.2 × 10^−3^), progranulin (16.3 × 10^−3^), clusterin (29.6 × 10^−3^), PEDF (42.3 × 10^−3^), or CTRP-3 (110 × 10^−3^). Based on these calculations, the high basal permeability of the BBB for METRNL explains the high concentrations of this adipo-myokine in CSF, which are similar to the respective serum concentrations. It is of high clinical interest why the myokine METRNL has such a high BBB permeability when compared to classical adipokines mentioned above. One explanation could be the fact that METRNL is a relatively small peptide that does not undergo an assembly into higher order molecular structures (dimeric, trimerci) as it was described for adiponectin or CTRP-3. Since the expression and secretion of METRNL strongly increases during and after muscle contraction or exercise [19,23,24,25], one could assume that even higher quantities of METRNL cross over the BBB than in the resting state. If this hypothesis is true, exercise-induced METRNL release might have the capability to directly modulate brain function and/or brain plasticity. This could open a new window of clinical research on the *myocyte–brain axis*.

Moreover, the inducible permeability of the BBB for METRNL was shown by the highly significant correlation of the widely used albumin CSF/serum ratio with CSF METRNL concentrations. In addition, the albumin CSF/serum ratio was also significantly correlated with the METRNL CSF/serum ratio as shown in our classical *Reibergrams*. Finally, a detailed laboratory grading system (0–3) was used for a specific description of the BBB. We found a highly significant increase in CSF METRNL levels along with increasing grades of BBB dysfunction and with rising CSF cell count (leukocytes, immune cells, lymphocytes). If BBB permeability increases (with increasing permeation of albumin as a general predictor), METRNL permeability also increases in parallel. Taken together, these results are novel and prove that METRNL fulfils the basal criteria of a novel and putative mediator of the *muscle–brain axis*.

In general, a putative autochthonous production of METRNL in cells of the central nervous system such as microglial cells has to be considered; however, data are extremely scarce. Activated microglial cells play a role in cytotoxic and inflammatory processes of neurodegenerative diseases as well as during aging. We remember that CSF METRNL levels were associated with increasing age in the present study. Although cells of the central nervous system might be able to express METRNL, a significant autochthonous production in CSF by ependymal/microglial cells is not probable on the basis of the present data and the CSF/serum ratios. A relevant autochthonous production could not explain the clear correlations of CSF levels with the serum concentrations and the albumin ratio. As a first step to detect any implications of METRNL concentrations for neurological diseases, we investigated 7 disease groups/entities. The limitations of the observational character of this study have to be kept in mind. METRNL serum or CSF concentrations were not altered in disease subgroups. However, future prospective studies are necessary to prove or to exclude the role of serum or CSF METRNL concentrations as a biomarker for disease. The limitation of this pilot study is given by the wide variety of neurological disease subgroups. The results are encouraging, but it is necessary to conduct prospective and confirmatory studies on more homogenous cohorts of neurological patients on the one hand, and on cohorts of patients with specific diseases (such as multiple sclerosis) on the other hand.

## 5. Conclusions

The present study provides, for the first time, a systematic quantification and biostatistical analysis of paired serum and CSF concentrations of the neurotrophic adipo-myokine METRNL. This work could provide a basis for the development of human reference ranges and for the establishment of disease-specific biomarkers. The blood–brain barrier was shown to be permeable for METRNL under basal and under inducible conditions when an increasing blood–brain barrier dysfunction causes increasing CSF METRNL concentrations. METRNL represents a novel and promising neurotrophic adipo-myokine and a putative mediator of the *muscle–brain axis*.

## Figures and Tables

**Table 1:** Characteristics of the study population (*n* = 260 individuals). Anthropometric and laboratory parameters in serum and CSF (cerebrospinal fluid) are shown. General patient characteristics have been published earlier by our group in several studies on the role of classical adipokines in CSF (1,6–8). Mean values + standard deviation (SD) and range are given for numerical parameters. For classified variables, absolute numbers and percentages are shown. The *p* values were calculated by using the *Mann–Whitney U* test for two subgroups (* *p* < 0.05). BMI, body mass index; CSF, cerebrospinal fluid; CNS, central nervous system; METRNL, Meteorin-like.

Study Population (*n* = 260)	
Age (years) [range]	50.5 ± 17.5 [18–90]
Males *n* (%)	107 (41.2)
Females *n* (%)	153 (58.8)
**Meteorin-like**	
METRNL in serum (pg/mL) [range]	801.2 ± 378.3 [203.1–2685.1]
METRNL in cerebrospinal fluid (pg/mL) [range]	1007.2 ± 624.2 [230.0–4275.3]
METRNL CSF/serum ratio [range]	1.4 ± 0.8 [0.2–6.7]
**Anthropometric parameters**	
Mean BMI (kg/m²) °	26.5 ± 5.0 [17.4–47.7]
BMI < 25.0 kg/m² *n* (%)	126 (48.5)
BMI ≥ 25.0 kg/m² *n* (%)	128 (49.2)
**Neurological diseases/Clinical subgroups**	
Infectious CNS disease *n* (%)	13 (5.0)
Multiple sclerosis *n* (%)	40 (15.4)
Vascular disease *n* (%)	22 (8.5)
Epilepsie *n* (%)	26 (10.0)
Headache/facial pain *n* (%)	23 (8.8)
Neuropathy/cranial nerve palsy *n* (%)	46 (17.7)
Others * *n* (%)	90 (34.6)
* consisting of dementia (8), psychiatric disorders (12), normal pressure hydrocephalus (8), and patients undergoing spinal puncture for exclusion of other diseases (53)	
**Serum METRNL**	
Males	745.3 ± 350.9 *
Females	840.2 ± 392.8 * (*p* = 0.031)
BMI < 25	774.7 ± 343.8
BMI ≥ 25	827.3 ± 411.3
Diabetes mellitus	945.0 ± 565.3
Non-Diabetes	784.3 ± 343.6
Hypertension	835.9 ± 433.7
Normotension	778.5 ± 330.5
Smoker	783.2 ± 320.9
Non-Smoker	807.1 ± 389.9
**CSF METRNL**	
Males	1071.2 ± 603.4
Females	962.4 ± 636.5
BMI < 25	970.9 ± 637.4
BMI ≥ 25	1041.6 ± 606.8
Diabetes mellitus	1128.1 ± 569.1
Non-Diabetes	993.9 ± 630.7
Hypertension	1117.5 ± 673.1 *
Normotension	929.6 ± 575.2 * (*p* = 0.023)
Smoker	915.5 ± 447.0
Non-Smoker	1029.7 ± 655.5

° BMI was not available in 2.3% of patients.

**Table 2:** Correlation analysis by *Spearman rho* test of serum or CSF METRNL concentrations with standard serum and CSF parameters. ALT, alanine-aminotransferase; AST, aspartate-aminotransferase; BMI, body mass index; CSF, cerebrospinal fluid; HDL, high density lipoprotein; LDL, low density lipoprotein; METRNL, Meteorin-like protein; n. s., not significant; Ig, immunoglobulin.

	Serum METRNL [pg/mL]	CSF METRNL [pg/mL]
**Correlation with**		
Age [years]	n. s.	rho = +0.237 *p* < 0.001
BMI [kg/m^2^)	n. s.	n. s.
**Serum parameters**		
METRNL [pg/mL]	-	rho = +0.521 *p* < 0.001
Leukocyte [giga/L]	n. s.	n. s.
Hemoglobin [g/L]	rho = −0.231 *p* < 0.001	n. s.
CRP [mg/dL]	rho = +0.159 *p* = 0.011	n. s.
ALT [U/L]	n. s.	rho = +0.169 *p* = 0.009
AST [U/L]	n. s.	rho = +0.255 *p* < 0.001
Creatinine [mg/dL]	n. s.	rho = -0.151 *p* = 0.016
Urea [mg/dL]	n. s.	rho = +0.142 *p* = 0.025
Albumin [g/L]	rho = −0.172 *p* = 0.005	n. s.
CSF/serum albumin ratio	n. s.	rho = +0.480 *p* < 0.001
Lipoprotein-Metabolism (triglycerides, LDL cholesterol, HDL cholesterol)	n. s.	n. s.
Carbohydrate-Metabolism (Glucose or HbA1c)	n. s.	n. s.
**CSF parameters**		
Total protein [g/L]	n. s.	rho = +0.420 *p* < 0.001
Albumin [g/L]	n. s.	rho = +0.463 *p* < 0.001
IgM [g/L]	n. s.	rho = +0.349 *p* = 0.003
IgG [g/L]	n. s.	rho = +0.357 *p* < 0.001
IgA [g/L]	rho = +0.348 *p* = 0.003	rho = +0.430 *p* < 0.001
Lactate [mmol/L]	n. s.	rho = +0.210 *p* = 0.001

**Table 3:** Serum concentrations, CSF concentrations, and CSF/serum (C/S) ratios for METRNL (Meteorin-like protein) in subgroups of disease entities and in BBB dysfunction. *p*-values were calculated applying the *Mann–Whitney U*-test for two subgroups and the *Kruskal–Wallis* test for more than two subgroups. BBB, blood–brain barrier.

	Serum METRNL [pg/mL]	CSF METRNL [pg/mL]	C/S METRNL
**Disease groups**			
Infectious disease (*n* = 13)	935.2 ± 434.6	1674.8 ± 1072.0	1.8 ± 0.8
Multiple Sclerosis (*n* = 40)	759.1 ± 367.3	909.2 ± 537.1	1.3 ± 0.7
Vascular disease (*n* = 22)	640.2 ± 391.6	879.5 ± 599.1	1.4 ± 0.6
Epilepsy (*n* = 26)	758.5 ± 292.8	927.6 ± 485.2	1.4 ± 1.0
Headache/facial pain (*n* = 23)	732.5 ± 312.6	864.0 ± 450.8	1.2 ± 0.4
Neuropathy/ cranial nerve palsy (*n* = 46)	800.3 ± 347.2	1068.7 ± 560.4	1.4 ± 0.6
Others (*n* = 90)	870.2 ± 412.6	1013.7 ± 640.3	1.3 ± 0.9
**BBB dysfunction**			
Grade 0 (*n* = 206)	800.4 ± 375.5	912.1 ± 545.9	1.2 ± 0.7
Grade 1 (*n* = 21)	765.4 ± 321.8	1151.5 ± 588.3	1.6 ± 0.7
Grade 2 (*n* = 31)	827.9 ± 431.8	1446.6 ± 749.3	1.9 ± 0.8
Grade 3 (*n* = 2)	842.3 ± 672.1	2473.9 ± 1857.2	3.0 ± 0.2
		*p* < 0.001	*p* < 0.001
**CSF cell count/µL**			
0–5 cells (*n* = 226)	784.4 ± 376.8	959.5 ± 561.7	1.4 ± 0.8
>5 cells (*n* = 34)	912.9 ± 374.7	1324.6 ± 887.4	1.4 ± 0.7
	*p* = 0.028	*p* = 0.035	
**Oligoclonal bands**			
negative (*n* = 207)	791.8 ± 375.4	1010.6 ± 609.1	1.4 ± 0.8
positive (*n* = 41)	806.9 ± 385.2	1070.9 ± 715.4	1.4 ± 0.7

## Data Availability

The data presented in this study are available on request from the corresponding author.

## References

[1] Hopfinger A., Berghoff M., Karrasch T., Schmid A., Schaffler A. (2021). Systematic quantification of neurotrophic adipokines rbp4, pedf, and clusterin in human cerebrospinal fluid and serum. J. Clin. Endocrinol. Metab..

[2] Ahima R.S., Saper C.B., Flier J.S., Elmquist J.K. (2000). Leptin regulation of neuroendocrine systems. Front. Neuroendocrinol..

[3] McMillen I.C., Adam C.L., Muhlhausler B.S. (2005). Early origins of obesity: Programming the appetite regulatory system. J. Physiol..

[4] Schnabl K., Li Y., Klingenspor M. (2020). The gut hormone secretin triggers a gut-brown fat-brain axis in the control of food intake. Exp. Physiol..

[5] De Kloet A.D., Herman J.P. (2018). Fat-brain connections: Adipocyte glucocorticoid control of stress and metabolism. Front. Neuroendocrinol..

[6] Berghoff M., Hochberg A., Schmid A., Schlegel J., Karrasch T., Kaps M., Schaffler A. (2016). Quantification and regulation of the adipokines resistin and progranulin in human cerebrospinal fluid. Eur. J. Clin. Invest..

[7] Neumeier M., Weigert J., Buettner R., Wanninger J., Schaffler A., Muller A.M., Killian S., Sauerbruch S., Schlachetzki F., Steinbrecher A. (2007). Detection of adiponectin in cerebrospinal fluid in humans. Am. J. Physiol. Endocrinol. Metab..

[8] Schmid A., Berghoff M., Hochberg A., Schaffler A., Karrasch T. (2017). Ctrp-3 is permeable to the blood-brain barrier and is not regulated by glucose or lipids in vivo. Eur. J. Clin. Invest..

[9] Giudice J., Taylor J.M. (2017). Muscle as a paracrine and endocrine organ. Curr. Opin. Pharmacol..

[10] Kirk B., Feehan J., Lombardi G., Duque G. (2020). Muscle, bone, and fat crosstalk: The biological role of myokines, osteokines, and adipokines. Curr. Osteoporos. Rep..

[11] Pedersen B.K. (2013). Muscle as a secretory organ. Compr. Physiol..

[12] Yoon J.H., Kim J., Song P., Lee T.G., Suh P.G., Ryu S.H. (2012). Secretomics for skeletal muscle cells: A discovery of novel regulators?. Adv. Biol. Regul..

[13] Weigert C., Hartwig S., Lehr S. (2021). Methods for proteomics-based analysis of the human muscle secretome using an in vitro exercise model. Methods Mol. Biol..

[14] Tok O., Kisioglu S.V., Ersoz H.O., Kahveci B., Goktas Z. (2021). Effects of increased physical activity and/or weight loss diet on serum myokine and adipokine levels in overweight adults with impaired glucose metabolism. J. Diabetes Complicat..

[15] Townsend L.K., Knuth C.M., Wright D.C. (2017). Cycling our way to fit fat. Physiol. Rep..

[16] Rao R.R., Long J.Z., White J.P., Svensson K.J., Lou J., Lokurkar I., Jedrychowski M.P., Ruas J.L., Wrann C.D., Lo J.C. (2014). Meteorin-like is a hormone that regulates immune-adipose interactions to increase beige fat thermogenesis. Cell.

[17] Lee J.O., Byun W.S., Kang M.J., Han J.A., Moon J., Shin M.J., Lee H.J., Chung J.H., Lee J.S., Son C.G. (2020). The myokine meteorin-like (metrnl) improves glucose tolerance in both skeletal muscle cells and mice by targeting ampkalpha2. FEBS J..

[18] Eaton M., Granata C., Barry J., Safdar A., Bishop D., Little J.P. (2018). Impact of a single bout of high-intensity interval exercise and short-term interval training on interleukin-6, fndc5, and metrnl mrna expression in human skeletal muscle. J. Sport Health Sci..

[19] Ost M., Coleman V., Kasch J., Klaus S. (2016). Regulation of myokine expression: Role of exercise and cellular stress. Free Radic. Biol. Med..

[20] Jung T.W., Lee S.H., Kim H.C., Bang J.S., Abd El-Aty A.M., Hacimuftuoglu A., Shin Y.K., Jeong J.H. (2018). Metrnl attenuates lipid-induced inflammation and insulin resistance via ampk or ppardelta-dependent pathways in skeletal muscle of mice. Exp. Mol. Med..

[21] Ushach I., Arrevillaga-Boni G., Heller G.N., Pone E., Hernandez-Ruiz M., Catalan-Dibene J., Hevezi P., Zlotnik A. (2018). Meteorin-like/meteorin-beta is a novel immunoregulatory cytokine associated with inflammation. J. Immunol..

[22] Wen D., Xiao Y., Vecchi M.M., Gong B.J., Dolnikova J., Pepinsky R.B. (2017). Determination of the disulfide structure of murine meteorin, a neurotrophic factor, by lc-ms and electron transfer dissociation-high-energy collisional dissociation analysis of proteolytic fragments. Anal. Chem..

[23] Bae J.Y. (2018). Aerobic exercise increases meteorin-like protein in muscle and adipose tissue of chronic high-fat diet-induced obese mice. Biomed. Res. Int..

[24] Bae J.Y., Woo J., Kang S., Shin K.O. (2018). Effects of detraining and retraining on muscle energy-sensing network and meteorin-like levels in obese mice. Lipids Health Dis..

[25] Huh J.Y. (2018). The role of exercise-induced myokines in regulating metabolism. Arch. Pharm. Res..

